# The mitochondrial genome of *Caenis* sp. (Ephemeroptera: Caenidae) from Fujian and the phylogeny of Caenidae within Ephemeroptera

**DOI:** 10.1080/23802359.2019.1698986

**Published:** 2019-12-12

**Authors:** Xiao-Dong Xu, Yi-Yang Jia, Xin-Yi Dai, Jin-Liang Ma, Kenneth B. Storey, Jia-Yong Zhang, Dan-Na Yu

**Affiliations:** a College of Chemistry and Life Science, Zhejiang Normal University, Jinhua, China;; b Key Lab of Wildlife Biotechnology, Conservation and Utilization of Zhejiang Province, Zhejiang Normal University, Jinhua, China;; c Department of Biology, Carleton University, Ottawa, Canada

**Keywords:** Ephemeroptera, Caenidae, mitochondrial genome, phylogeny

## Abstract

The phylogenetic relationship of Caenidae remains hotly debated within the Ephemeroptera. We sequenced the complete mitochondrial genome of *Caenis* sp. (Ephemeroptera: Caenidae) to discuss the phylogenetic relationships among the Caenidae. The mitochondrial genome of *Caenis* sp. collected from Jian’ou, Fujian province, China is a circular molecule of 15,392 bp in length containing 37 genes (13 protein-coding genes, 22 tRNAs, and two rRNAs), which showed the typical insect mitochondrial gene arrangement. In BI and ML phylogenetic trees using 23 species from 13 families, the monophyly of the families Caenidae, Heptageniidae, Isonychiidae, and Vietnamellidae was strongly supported. The clade of Caenidae is a sister clade to the clade of Teloganodidae and Baetidae.

The phylogenetic relationships among the Ephemeroptera has always been a research hotspot (Hebert et al. 2003; Ogden and Whiting 2005; Sun et al. 2006; O’Donnell and Jockusch 2008; Ogden et al. 2009; Webb et al. 2012; Saito et al. 2016; Cai et al. 2018; Gao et al. 2018; Ye et al. 2018). However, there are relatively few studies of the phylogenetic relationships within the Caenidae. Ogden and Whiting (2005) showed the monophyly of Caenoidea (Caenidae + Neoephemeridae) when using *12S rDNA*, *16S rDNA*, *18S rDNA*, *28S rDNA*, and *H3* gene sequences of Ephemeroptera species to explore the phylogenesis of this group. Ye et al. (2018) and Cai et al. (2018) supported the monophyly of Caenidae. More molecular evidence needs to be discovered to clarify the monophyly of Caenidae. Thus, we sequenced the mitochondrial genome of *Caenis* sp. to further assess the phylogenetic relationship of Caenidae within Ephemeroptera.

The samples of *Caenis* sp. were collected from Jianxi River, Jian’ou (N 27.019°, E 118.304°), Fujian Province, China. The sample (CA20180809) was identified and stored at −40 °C in the Animal Specimen Museum, College of Life Sciences and Chemistry, Zhejiang Normal University, China. Total genomic DNA was extracted from individual tissues of the sample using Ezup Column Animal Genomic DNA Purification Kit (Sangon Biotech Company, Shanghai, China) and stored in the Zhang laboratory. Universal primers were used to amplify some partial fragments as described in Zhang et al. (2008). Subsequently, the remaining gaps were sequenced by utilizing species-specific primers according to previously obtained sequences. The mitochondrial genome is deposited in GenBank with accession number MN356096.

The complete mitochondrial genome of *Caenis* sp. was a typical circular DNA molecule of 15,392 bp in length. The AT content of the whole genome and the control region was 71.5% and 73.2%, respectively. All 13 PGCs began with ATN (N represents A, T, G, and C) as the start codon. The *ATP6*, *ATP8*, *COIII*, *ND2*, *ND3*, *ND4L*, *ND5*, and *ND6* genes were terminated with TAA or TAG as the stop codon. *ND1* ended with TAG and the other PGCs (*COI*, *COII*, *ND4*, *Cyt b*) ended with the incomplete stop codon T––.

The phylogenetic relationship was constructed from the 13 PCGs using two methods: Bayesian inference (BI) using MrBayes 3.1.2 (Huelsenbeck and Ronquist 2001) and maximum-likelihood (ML) using RAxML 8.2.0 (Stamatakis 2014). The mitochondrial genomes of 22 Ephemeroptera species downloaded from GenBank (Zhang et al. 2008; Li et al. 2014; Tang et al. 2014; Zhou et al. 2016; Cai et al. 2018; Gao et al. 2018; Ye et al. 2018) were used to investigate the phylogenetic relationships. *Siphluriscus chinensis* was used as the outgroup. Each alignment was performed using Gblock 0.91 b (Castresana 2000) using default settings, beneficial to select conserved regions of the nucleotide. The monophyly of the families Heptageniidae, Isonychiidae, Vietnamellidae, and Caenidae was strongly supported in both BI and ML analyses (Figure 1). Isonychiidae was the basal clade to Ephemeroptera excluding the outgroup Siphluriscidae. Caenidae was a sister clade to the clade of Baetidae and Teloganodidae.

**Figure 1.**
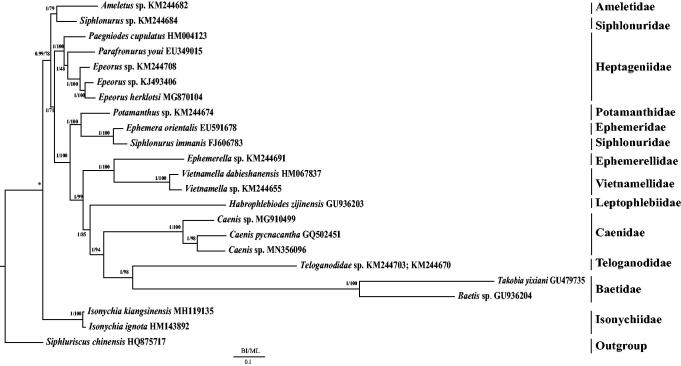
Phylogenetic tree of the relationships among 23 species of Ephemeroptera, including *Caenis* sp. (MN356096) based on the nucleotide dataset of the 13 mitochondrial protein-coding genes. *Siphluriscus chinensis* was used as the outgroup. The numbers above branches specify posterior probabilities as determined from BI (left) and bootstrap percentages from ML (right). The GenBank accession numbers of all species are shown in the figure.
